# Cardiovascular Risk Factor Burden in Africa and the Middle East: The Africa Middle East Cardiovascular Epidemiological (ACE) Study

**DOI:** 10.1371/journal.pone.0102830

**Published:** 2014-08-04

**Authors:** Alawi A. Alsheikh-Ali, Mohamed I. Omar, Frederick J. Raal, Wafa Rashed, Omar Hamoui, Abdoul Kane, Mohamed Alami, Paula Abreu, Walid M. Mashhoud

**Affiliations:** 1 Institute of Cardiac Sciences, Sheikh Khalifa Medical City, Abu Dhabi, United Arab Emirates; 2 Pfizer AfME, Dubai Media City, Dubai, United Arab Emirates; 3 Department of Medicine, Faculty of Health Sciences, Johannesburg Hospital, Johannesburg, South Africa; 4 Mubarak Al Kabeer Hospital, Al Jabriya, Kuwait; 5 Clemenceau Medical Center, Beirut, Lebanon; 6 L'Hopital General de Grand Yoff, Dakar, Senegal; 7 Mohammed V University, Rabat, Morocco; 8 Pfizer Inc., New York, New York, United States of America; 9 Pfizer Saudi Arabia, King's Tower, Jeddah, Saudi Arabia; University of Washington, United States of America

## Abstract

**Background:**

Increased urbanization in the developing world parallels a rising burden of chronic diseases. Developing countries account for ∼80% of global cardiovascular (CV) deaths, but contribute a paucity of systematic epidemiological data on CV risk factors.

**Objective:**

To estimate the prevalence of CV risk factors in rural and urban cohorts attending general practice clinics in the Africa and Middle East (AfME) region.

**Methods:**

In a cross-sectional epidemiological study, the presence of CV risk factors (hypertension, diabetes mellitus (diabetes), dyslipidemia, obesity, smoking and abdominal obesity) were evaluated in stable adult outpatients attending general practice primary care clinics. A rural population was defined as isolated (>50 km or lack of easy access to commuter transportation) from urban centers.

**Results:**

4,378 outpatients were systematically recruited from 94 clinics across 14 AfME countries. Mean age was 46±14 years and 52% of outpatients were female. A high prevalence of dyslipidemia (70%) and abdominal obesity (68%) were observed, followed by hypertension (43%) and diabetes (25%). The vast majority of outpatients (92%) had at least one modifiable CV risk factor, many (74%) had more than one, and half (53%) had 3 or more. These findings were observed in both genders and across urban and rural centers. Among outpatients with pre-existing hypertension or dyslipidemia, many were not at their target blood pressure or LDL-cholesterol goals.

**Conclusion:**

Cardiovascular risk factors are highly prevalent among relatively young, stable outpatients attending general practice clinics across AfME. The findings support opportunistic screening for CV risk factors whenever outpatients visit a general practitioner and provide an opportunity for early identification and management of CV risk factors, including lifestyle interventions.

## Introduction

Conventional risk factors for cardiovascular (CV) disease are becoming increasingly prevalent worldwide and underlie the growing global burden of non-communicable diseases [1], [2]. While developing countries are expected to account for 80% of global deaths from CV disease, little systematic epidemiological data on CV risk factors are available from these countries [3], [4]. Most prior studies from developing regions of the world predated recent socioeconomic developments, focused on specific countries, or enrolled outpatients from specialist or acute care settings [5]–[7].

Many countries in Africa and the Middle East (AfME), as in other developing regions, have undergone an epidemiological transition, experiencing significant urbanization in recent years [4], [5]. The proportion of individuals living in urban centers in the developing world has doubled between 1970 and 1994, and is expected to double again by 2025 [8]. This rapid urbanization has occurred in parallel with a rising burden of chronic diseases, but developments in national preventive health systems and screening programs have trailed behind. In the absence of an infrastructure for universal CV screening in many developing countries, targeted or opportunistic screening strategies may be useful alternatives. In particular, opportunistic screening targeted at adults attending general practice clinics may prove valuable for early detection of CV risk factors [9]–[11].

The Africa Middle East Cardiovascular Epidemiological (ACE) Study is a multinational cross-sectional study to determine the prevalence of CV risk factors in outpatients attending urban and rural general practice clinics in the AfME region.

## Methods

### Study Design and Objectives

The ACE Study was a cross-sectional epidemiological study conducted in 94 clinics across 14 countries in the AfME region between July 2011 and April 2012. In particular, the study was aimed at countries in the AfME region where there was a paucity of systematic epidemiological data. Furthermore, site selection was based upon the ability of a site to conduct clinical studies based on the availability of clinical research expertise, infrastructure and ethical oversight. The primary objective was to estimate the prevalence of CV risk factors in outpatients attending general practice and other non-specialist clinics in urban and rural communities. Rural areas were defined as those isolated from urban centers by a distance of >50 km, or those with a lack of easy access to commuter transportation [12]. In outpatients with a pre-existing diagnosis of hypertension or dyslipidemia, the degree of control of these risk factors was assessed.

### Subject Selection

Male and female outpatients >18 years of age were enrolled after signing an informed consent form. Pregnant women, lactating mothers, and outpatients with life-threatening conditions were excluded. To prevent selection bias, every fifth outpatient seen by a physician or general practitioner on a particular day and fulfilling the inclusion/exclusion criteria was enrolled. The physicians responsible evaluated outpatients through history taking, physical examination, and laboratory investigations. Evaluations were typically undertaken over one clinic visit; however, for non-fasting outpatients during the first visit, a second visit was arranged to obtain fasting blood samples.

### Definitions

Dyslipidemia was recorded if the outpatient was on treatment with lipid-regulating drugs or if a current fasting lipid profile measurement documented one or more of the following: high low density lipoprotein (LDL)-cholesterol; high total cholesterol; low high density lipoprotein (HDL)-cholesterol; or high triglyceride level according to the National Cholesterol Education Program (NCEP) guidelines [13]. Outpatients on lipid-regulating treatments were considered to have controlled LDL-cholesterol levels if their values were at goal according to their risk category, based on the NCEP recommended LDL-cholesterol targets [13].

Arterial blood pressure was recorded as the higher of two consecutive measurements, taken once in each arm with a standardized blood pressure measuring instrument after the outpatient had been sitting quietly for at least 5 minutes. Hypertension was defined as being on current antihypertensive drugs, or having an abnormal blood pressure reading according to the European Society of Cardiology (ESC) Cardiovascular Prevention Guidelines [14]. Outpatients on antihypertensive drugs were considered to have controlled blood pressure if they had values below the targets set by the ESC guidelines [14].

The following modifiable CV risk factors were also recorded: diabetes mellitus (diabetes) defined as per the American Diabetes Association criteria [15]; smoking defined as current or past consumption of cigarettes, pipe or water pipe (shisha); obesity defined as body mass index (BMI) ≥30 kg/m^2^; and abdominal obesity defined in-line with the International Diabetes Federation (IDF) harmonized criteria as a waist circumference ≥94 cm in a man and ≥80 cm in a woman [16].

### Statistical Methods

Assuming that at least 90% of enrolled outpatients would contribute to the analysis, a planned sample size of 4,300 permitted reasonably precise estimates of the percentage of outpatients with dyslipidemia or hypertension to within ±1.6% with 95% confidence. Categorical data are summarized using percentages and 95% confidence intervals. Continuous data are reported using n, mean ± standard deviation or median [25^th^, 75^th^ percentiles] as appropriate. All statistical tests were two-sided and *p*-values <0.05 were considered statistically significant.

Ethics approval was obtained from all participating centers and appropriate regulatory bodies in each country, and the study was registered on clinicaltrials.gov (registration number NCT01243138).

## Results

**Figure 1:**
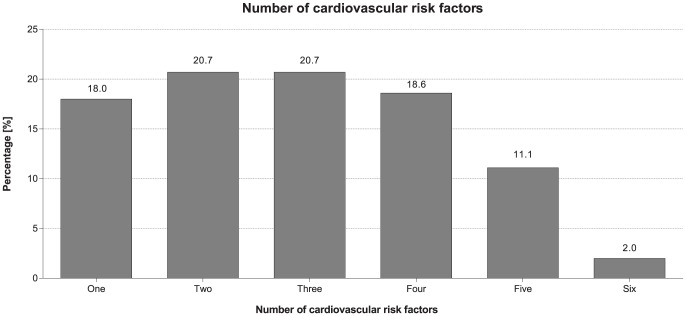
Proportion of outpatients presenting with one or more cardiovascular risk factors. Graph to show the percentage of outpatients presenting with 1–6 CV risk factors. Risk factors: dyslipidemia prevalence, hypertension prevalence, obesity (defined by BMI ≥30 kg/m^2^) prevalence, abdominal obesity prevalence, diabetes prevalence, and smoking prevalence.

A total of 4,378 subjects from 94 outpatient general practice clinics in 14 countries (2,337 outpatients from eight countries in Africa, and 2,041 outpatients from six countries in the Middle East) were analyzed. Nearly one third (31%) of the total cohort were enrolled from centers in rural communities. The mean age was 46 years, with near-equal representation of genders. Almost half of the outpatients (46%) were <45 years old and only 10% were 65 years or older. The vast majority of outpatients (92%) had at least one of the six modifiable CV risk factors we measured: dyslipidemia, hypertension, diabetes, obesity (BMI ≥30 kg/m^2^), abdominal obesity and current smoking. Many (74%) outpatients had more than one risk factor: 18% had one risk factor, 21% had two risk factors, 21% had three, 19% had four, 11% had five, and 2% had six risk factors (Figure 1).

### Dyslipidemia: Prevalence and Risk Factor Control

**Figure 2:**
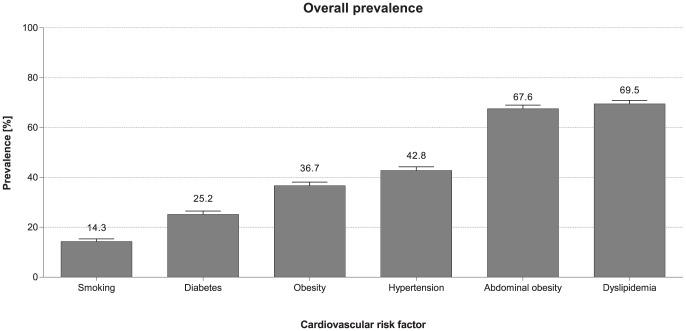
Overall prevalence of cardiovascular risk factors across all participating countries. Graph to show the overall prevalence and standard error of dyslipidemia, hypertension, obesity (defined by BMI ≥30 kg/m^2^), abdominal obesity, diabetes, and smoking across the AfME region.

**Figure 3:**
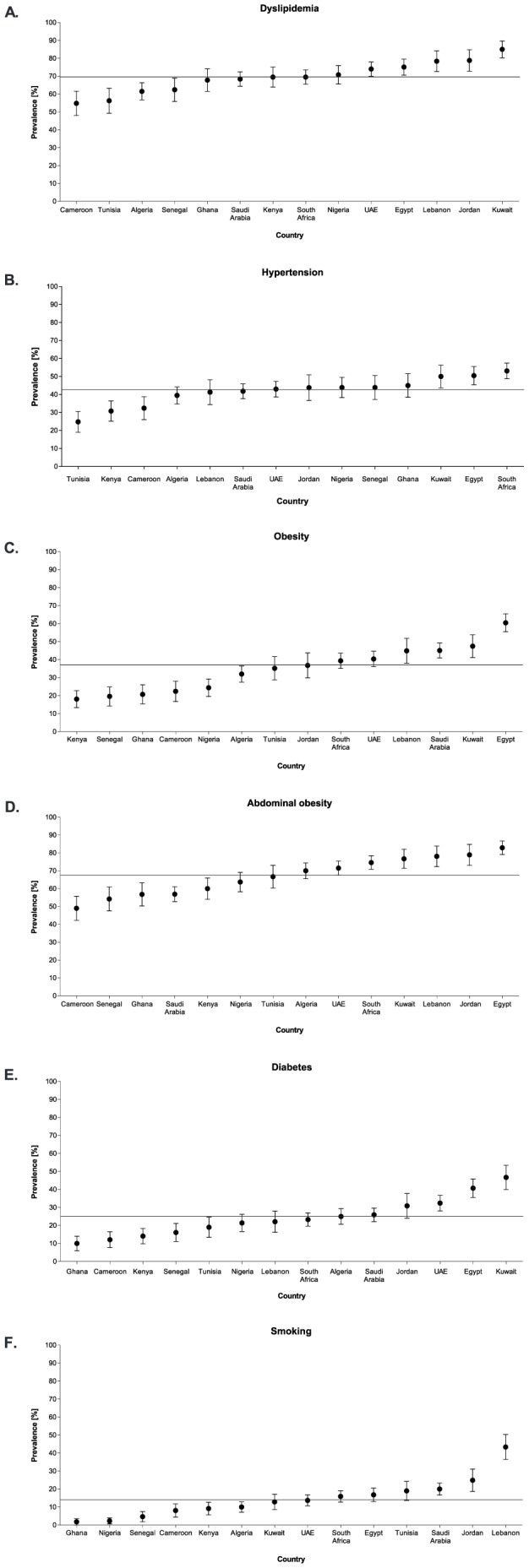
Prevalence data and standard error per participating country. Graphs depicting (A) dyslipidemia prevalence, (B) hypertension prevalence, (C) obesity (defined by BMI ≥30 kg/m^2^) prevalence, (D) abdominal obesity prevalence, (E) diabetes prevalence, and (F) smoking prevalence. Overall prevalence is shown by a solid line.

The median [25^th^, 75^th^ percentiles] lipid values in the overall cohort were 185 [158, 214], 112 [89, 140], 46 [37, 55], and 88 [51, 139] mg/dL for total cholesterol, LDL-cholesterol, HDL-cholesterol, and triglycerides, respectively (Table 1). Dyslipidemia was the most prevalent CV risk factor recorded in nearly three out of every four outpatients (70%) (Figure 2). The prevalence of dyslipidemia exceeded 50% in all countries and ranged from 55% in Cameroon to 85% in Kuwait (Figure 3A). Only 16% of the outpatients were on a prior lipid-altering drug, predominantly a statin. The most common component of dyslipidemia was low HDL-cholesterol, recorded in nearly 30% of the whole study cohort. Many outpatients were not at their LDL-cholesterol goals, particularly those in a high CV risk category as defined by NCEP [13] (11%, 34% and 79% not at LDL-cholesterol goal among low-risk, moderate-risk and high-risk outpatients, respectively). Overall, for almost every two outpatients without a prior diagnosis of dyslipidemia, screening at study encounter identified one new dyslipidemia diagnosis (2,131 outpatients; 48%).

**Table 1:** Median baseline parameters across the total cohort and male and female populations.

Variable	Total cohort	Male	Female
**Age (N) (%)**			
18–44 years	2,013 (46.0)	928 (45.0)	1,070 (46.8)
45–64 years	1,930 (44.1)	930 (45.1)	988 (43.2)
≥65 years	425 (9.7)	202 (9.8)	221 (9.7)
Mean years (range)	46.0 (18–110)	46.6 (18–110)	45.6 (18–89)
**Systolic blood pressure (mmHg)**			
Median (25^th^, 75^th^ percentile)	130.0 (120.0, 140.0)	130.0 (120.0, 140.0)	130.0 (120.0, 140.0)
**Diastolic blood pressure (mmHg)**			
Median (25^th^, 75^th^ percentile)	80.0 (74.0, 90.0)	80.0 (75.0, 90.0)	80.0 (73.0, 90.0)
**Waist circumference (cm)**			
Median (25^th^, 75^th^ percentile)	94.0 (85.0, 104.0)	94.0 (86.0, 103.1)	94.0 (84.0, 104.0)
**BMI (kg/m^2^)**			
Median (25^th^, 75^th^ percentile)	28.0 (24.5, 32.0)	27.2 (24.0, 30.7)	29.0 (24.9, 33.3)
**Total cholesterol (mg/dL)**			
Median (25^th^, 75^th^ percentile)	185.3 (158.3, 214.0)	181.5 (154.0, 209.0)	189.0 (162.2, 218.5)
**LDL-C (mg/dL)**			
Median (25^th^, 75^th^ percentile)	112.0 (88.8, 140.0)	110.0 (86.0, 138.0)	115.0 (91.0, 142.0)
**HDL-C (mg/dL)**			
Median (25^th^, 75^th^ percentile)	46.0 (37.0, 55.0)	42.5 (34.7, 52.0)	48.0 (40.0, 58.0)
**Triglycerides (mg/dL)**			
Median (25^th^, 75^th^ percentile)	88.0 (50.6, 139.0)	97.5 (57.0, 151.0)	79.0 (46.3, 125.5)
**Fasting plasma glucose (mmol/L)**			
Median (25^th^, 75^th^ percentile)	5.3 (4.8, 6.2)	5.4 (4.9, 6.3)	5.2 (4.7, 6.1)

### Hypertension: Prevalence and Risk Factor Control

The median [25^th^, 75^th^ percentiles] systolic and diastolic blood pressure values were 130 [120, 140] and 80 [74, 90] mmHg, respectively (Table 1). Hypertension was recorded in 43% of outpatients (Figure 2); 33% based on present therapy with antihypertensive drugs and an additional 10% based on an abnormal blood pressure reading at study encounter. Among outpatients with an abnormal blood pressure reading at study entry, 25% had an isolated elevation in systolic blood pressure, 15% had an isolated elevation in diastolic blood pressure, and 60% had combined systolic and diastolic abnormal readings. Hypertension was recorded in at least one quarter of enrolled outpatients in each country and ranged from 25% in Tunisia to 53% in South Africa (Figure 3B). Of the 1,437 outpatients with a pre-existing diagnosis of hypertension and on antihypertensive therapy at study entry, more than half (53%) had blood pressure readings above their target goals as set by the ESC prevention guidelines [14]. Overall, for every 10 outpatients without a prior diagnosis of hypertension, screening at study encounter identified one outpatient with an elevated blood pressure (434 outpatients; 10%).

### Obesity: Prevalence

The median waist circumference was 94 [86, 103.1] cm in men and 94 [84, 104] cm in women. The median BMI was 28 [25], [32] kg/m^2^ (Table 1). The prevalence of obesity, as defined by waist circumference (i.e. abdominal obesity), was nearly twice as common compared to when defined by BMI ≥30 mg/m^2^ (68% *vs* 37%) (Figure 2). This was true across all countries, particularly in some sub-Saharan Africa countries (Kenya, Ghana, Senegal, and Nigeria) where the ratio of prevalence of abdominal obesity to obesity by BMI was nearly 3-fold. In each country, approximately one in two enrolled outpatients had abdominal obesity, with a prevalence ranging from 49% in Cameroon to 83% in Egypt (Figure 3D). Overall, two outpatients with abdominal obesity were identified for every three outpatients screened (2,961 outpatients; 68%) and one outpatient with obesity by BMI ≥30 kg/m^2^ for approximately every three screened (1,606 outpatients; 37%).

### Diabetes: Prevalence

The median [25^th^, 75^th^ percentile] fasting plasma glucose level for the overall cohort was 5.3 [4.8, 6.2] mmol/L (Table 1). One quarter of the outpatients had diabetes (Figure 2) with 19% having a pre-existing diagnosis and an additional 5% diagnosed at the time of study entry based on a single fasting serum glucose of ≥7 mmol/L. There was considerable variation in the prevalence of diabetes by country, ranging from 10% in Ghana to 47% in Kuwait (Figure 3E). Overall, for every 20 outpatients without a prior diagnosis of diabetes, screening at study encounter uncovered one outpatient with a new diagnosis of diabetes (211 outpatients; 5%).

### Smoking: Prevalence

The prevalence of smoking was 25% (14% current smokers, and 11% past smokers) (Figure 2). The prevalence of current smoking ranged from 2% in Ghana to 43% in Lebanon (Figure 3F). Among current smokers, the majority (88%) reported smoking cigarettes and 1.2% reported pipe smoking (shisha).

### Prevalence of Risk Factors by Age, Gender and Community (Urban vs Rural)

**Figure 4:**
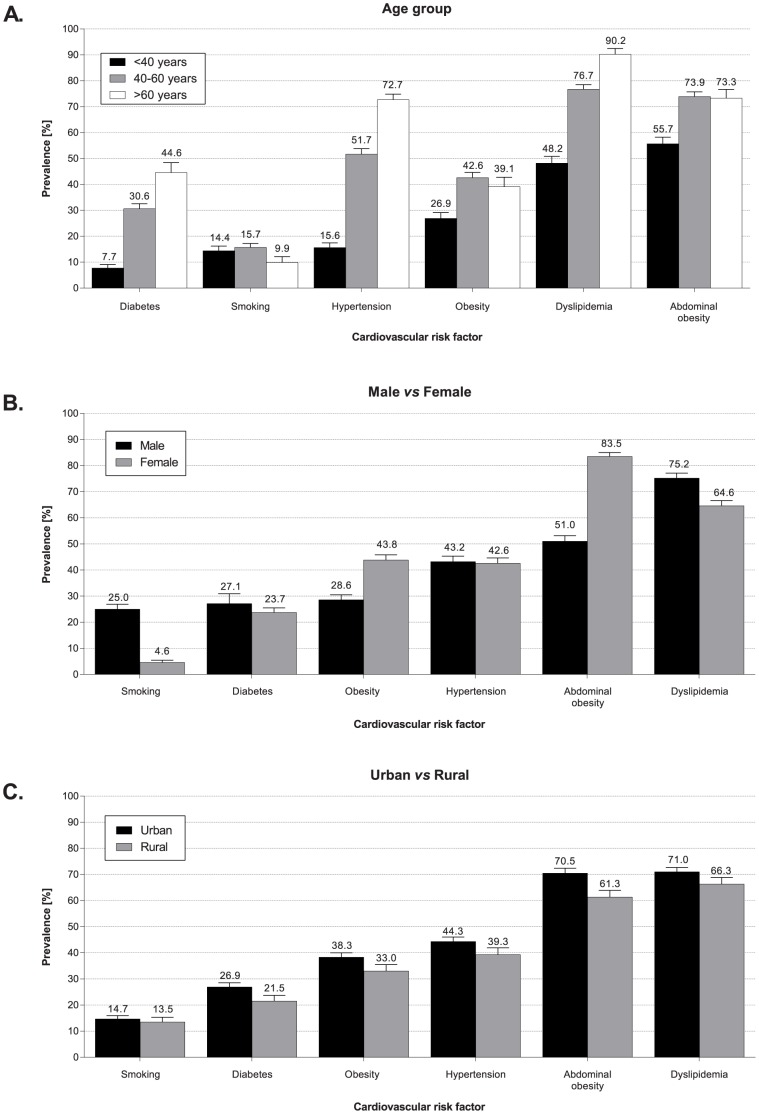
Overall cohort prevalence per investigated cardiovascular risk factor. Graphs to show the difference in total cohort prevalence data for each risk factor (dyslipidemia, hypertension, diabetes, obesity (defined by BMI ≥30 kg/m^2^), abdominal obesity, and smoking) by (A) age, (B) gender, and (C) location.

While older outpatients had numerically higher rates of dyslipidemia, hypertension, obesity, and diabetes, these conditions were also present in a large proportion of younger outpatients (e.g. ∼one in two outpatients <40 years with dyslipidemia or abdominal obesity) (Figure 4A). The prevalence of dyslipidemia, hypertension, and diabetes were comparable in males and females, with an apparent gender disparity in the prevalence of obesity and abdominal obesity (more common in females) and smoking (more common in males) (Figure 4B). While the prevalence estimates of most CV risk factors were higher in urban centers, rural centers still exhibited significant rates of dyslipidemia, hypertension, diabetes, and obesity, with estimates ranging from 22% of the outpatients affected by diabetes to 66% with dyslipidemia and 61% with abdominal obesity. Notably, smoking was equally prevalent in urban and rural communities (Figure 4C).

## Discussion

In this large contemporary cohort of adults attending general practice clinics across AfME, we observed alarming rates of CV risk factors. For every 10 outpatients we screened, nine had at least one conventional risk factor for CV disease. Dyslipidemia and abdominal obesity were the most prevalent factors, affecting approximately two thirds of screened outpatients, followed by high rates of hypertension, diabetes, and smoking. These findings were observed in both genders, across different age groups, and in urban as well as rural communities.

Of the 14 countries included in this study, Kuwait had the highest prevalence of dyslipidemia (85%). A previous study examining CV risk factors in Kuwait found prevalence of dyslipidemia to be lower at 73% [17]. However, outpatients in the previous study were aged between 20–65 years; higher prevalence in the present study may be due to the inclusion of outpatients over 65 years of age.

Diabetes prevalence for individual countries as estimated for 2013 by the IDF were consistently lower than those reported in this study (with the exception of Saudi Arabia, where a similar prevalence was observed in both studies) [18]. For example, in the present study the prevalence of diabetes in Cameroon was 12.1% in comparison to 4.9% as estimated by the IDF. The IDF data for Cameroon was estimated based on results from a survey carried out in 2003 and may indicate an increase in prevalence of diabetes over the past decade [19]. It is also possible that the patient population studied here (i.e. subjects attending outpatient clinics) represent a less healthy cohort with a higher prevalence of diabetes than the general population.

Primary prevention strategies including early detection and adequate control of conventional risk factors are critical to combating the global burden of CV disease [10], [20], [21]. In the Middle East the risk of acute myocardial infarction in the population can be almost entirely explained by nine modifiable risk factors, including those that were measured in this study [20]. In fact, the population-attributable risk for these factors was higher in the Middle East than in other regions of the world, highlighting the substantial benefits expected from aggressive preventive measures [22]. However, prevention is contingent on early detection, and most developing countries lack the necessary national infrastructure for comprehensive screening [23]. The results of our study provide a compelling rationale for opportunistic screening of CV risk factors as an alternative to more expensive and comprehensive population-wide screening. We show that opportunistic screening at the general practice consultation provides a high yield in identifying individuals at risk who would benefit from primary preventive measures.

The alarming burden of CV risk observed in the current study is particularly disturbing given the young age of the screened cohort; nearly half of the outpatients were <45 years of age, and 90% were <65 years. Several recent studies from the region have noted that patients with manifestations of CV disease, such as acute coronary syndrome or atrial fibrillation, are at least a decade younger than their counterparts in developed countries [24]–[27]. It is uncertain if these observations are entirely reflective of differences in population demographics (e.g. overall a younger population in developing countries) or instead the result of a predisposition to premature CV disease in the developing world [28]. Our findings of prevalent undiagnosed and uncontrolled risk factors in the young population of AfME is consistent with these observations [29] and highlights a pathophysiologic substrate for a predisposition to premature CV events.

Historically, disease profiles in societies have been linked to economic and social structures with developments in these societal aspects, such as the transition from rural to urban living, often linked to changes in disease patterns [30], [31]. As populations move from rural to urban settings, the burden of CV risk factors tends to increase [2], [32]. While we observed a similar general pattern of more prevalent risk factors in urban communities in the present study, the more notable observation in our opinion is the modest difference in the prevalence of most of these modifiable risk factors between urban and rural communities. Rural communities appear to be reaching prevalence rates comparable to their urban counterparts in terms of CV burden. Our study does not provide an explanation for the narrowing rural-urban gap in CV burden, but the adoption of an “urban” lifestyle by rural communities may be a contributing factor. Notably, in the current study, three out of every five outpatients in rural communities had abdominal obesity, and smoking was equally prevalent in rural and urban cohorts. Equal attention needs to be given to rural communities in the efforts to combat CV disease in the developing world [33], [34].

We observed an epidemic of obesity across the 14 countries we studied. This was true in both genders, across age groups, and in urban as well as rural communities. Obesity prevalence in Jordan was approximately equal to the mean value of all countries included in this study (36.8%); this level is lower than a previous study examining obesity in Jordan where prevalence was found to be 49.7% [35]. The prevalence of obesity was more striking when we used waist circumference definitions proposed by the IDF and other international organizations. Using a BMI definition underestimated the burden of obesity by nearly 50% and even 3-fold in some sub-Saharan countries. The limited value of BMI has been well documented in the multi-ethnic case-control study of acute myocardial infarction, INTERHEART [22], where waist and hip circumferences and their ratio were closely associated with risk of myocardial infarction even after adjustment for BMI and other risk factors. In the Middle East cohort of INTERHEART, abdominal obesity, not BMI, was significantly associated with the risk of myocardial infarction, accounting for ∼25% of the population-attributable risk [7]. The epidemic prevalence of this important risk factor highlights the need for innovative strategies in combating it. Interventional pharmacologic or surgical efforts, or sporadic weight loss campaigns, are unlikely to have a sustainable impact on the obesity burden [36]. Behavioral strategies focused at encouraging and sustaining a healthy and active lifestyle beginning in childhood are more promising [37]. Such strategies need to be supported by public policies, which define dietary intake, urban planning, and workplace environment to create sustainable healthy-heart communities [38], [39].

Our study is notable for focusing on a region with sparse systematic data on CV risk factors. The findings are strengthened by including a large number of outpatients from diverse countries in AfME, including countries in sub-Saharan Africa rarely included in such studies. More than 50% of the cohort was female, and a third of the cohort was from rural communities. As the cohort was recruited from a general practice/primary care setting, the results are more representative of the general population than studies previously focusing on inpatient or subspecialty settings. Nonetheless, the findings are limited by the cross-sectional design and obligatory reliance on one-time measurements of risk factors, as well as the lack of data on other variables such as social class and health insurance status that may affect CV risk. Future work should complement these findings with longitudinal and more comprehensive data. Furthermore, as this cohort has access to primary care, selection bias may exist since these outpatients may be more affluent in comparison to the general population.

The ACE study complements the existing literature on CV epidemiology in the developing world, and provides novel insights on the risk burden in the AfME region. Modifiable CV risk factors are highly prevalent among relatively young men and women attending general practice clinics across urban and rural communities in the 14 countries studied. The findings support opportunistic screening for CV risk when visiting a general practitioner, which provides an opportunity for early identification and management including lifestyle interventions. Urgent commitment from governments, policymakers, healthcare professionals and all other stakeholders towards CV prevention and promotion of healthy lifestyles is essential. Preventive strategies in this regard will be rewarded by an eventual decline in the rates of stroke and myocardial infarction that have become so prevalent in our communities.
